# Bayesian Analysis of the HR–VO_2_ Relationship during Cycling and Running in Males and Females

**DOI:** 10.3390/ijerph192416914

**Published:** 2022-12-16

**Authors:** Pat R. Vehrs, Nicole D. Tafuna’i, Gilbert W. Fellingham

**Affiliations:** 1 Department of Exercise Sciences, Brigham Young University, Provo, UT 84602, USA; 2 Department of Statistics, Brigham Young University, Provo, UT 84602, USA

**Keywords:** exercise prescription, Karvonen heart rate, exercise intensity, oxygen uptake

## Abstract

Professional organizations advise prescribing intensity of aerobic exercise using heart rate reserve (%HRR) which is presumed to have a 1:1 relationship with either maximal oxygen uptake (%VO_2_max) or %VO_2_ reserve (%VO_2_R). Even though running and cycling are popular modes of training, these relationships have not been investigated in a group of males and females during both running and cycling. This study evaluated the %HRR-%VO_2_max and %HRR–%VO_2_R relationships in 41 college-aged males (*n* = 21) and females (*n* = 20) during treadmill running and cycling. Heart rate (HR) and VO_2_ data were collected at rest and during maximal exercise tests on a treadmill and cycle ergometer. The HR and VO_2_ data were analyzed using a Bayesian approach. Both the %HRR-%VO_2_max and %HRR–%VO_2_R relationships did not coincide with the line of identity in males and females in both treadmill running and cycling. %HRR was closer to %VO_2_max than to %VO_2_R. There were no significant differences in the intercepts of the %HRR–%VO_2_max and %HRR–%VO_2_R relationships between males and females during running or cycling, or between running and cycling in males or females. The credible intervals of the intercepts and slopes suggest interindividual variability in the HR–VO_2_ relationship that would yield significant error in the prescription of intensity of aerobic exercise for an individual.

## 1. Introduction

There are an increasing number of positive health outcomes linked to physical activity [1,2]. The current recommendation is that adults should accrue at least 150 min to 300 min of moderate intensity physical activity per week, 75 min to 150 min of vigorous aerobic exercise per week, or an equivalent combination of the two [1,3,4]. Because of the dose–response relationship between exercise and cardiorespiratory fitness (CRF), greater volumes of exercise are necessary to further improve or maintain CRF [5]. The concept of a threshold intensity of exercise necessary for the improvement of CRF has long been suggested [6] and remains an important principle of the exercise prescription [3,7]. Incremental changes in the intensity of aerobic exercise are reflected in the heart rate (HR) and oxygen consumption (VO_2_) responses to exercise. Because the relationship between HR and VO_2_ during incremental aerobic exercise is considered to be linear [8], the intensity of aerobic exercise is typically expressed relative to maximal heart rate (HRmax), HR reserve (HRR), VO_2_max, or VO_2_ reserve (VO_2_R). The classification of intensity of aerobic exercise based on these methods is described by the American College of Sports Medicine (ACSM; [3,7]). The inherent problems with expressing intensity of aerobic exercise as a %HRmax or %VO_2_max have previously been described [9,10,11,12]. Briefly, there is an inequality between a given percentage of maximal HR and the equivalent percentage of VO_2_max due to disproportional non-zero values at rest. Using a percentage of reserve values to describe intensity of aerobic exercise corrects for non-zero resting values. The ACSM previously assumed that a given %HRR was equivalent to the same %VO_2_max [13,14]. Subsequent research has shown that the %HRR–%VO_2_max relationship does not coincide with the line of identity (x = y) and the %HRR–%VO_2_R relationship is indistinguishable from the line of identity in healthy [11] and obese [15] adults, elite cyclists [16], and during aerobic dance [17]. Other studies have reported that although the %HRR–%VO_2_R relationship did not coincide with the line of identity, there was a closer relationship between %HRR and %VO_2_R than between %HRR and %VO_2_max [18,19]. To the contrary, Gaskill et al. [20] reported that the slope and intercept for the relationship between %HRR and %VO_2_max were closer to the line of unity than the slope and intercept for the %HRR and %VO_2_R relationship. Although previous research is not in complete agreement about the HR–VO_2_ relationship, the ACSM now recognizes an equivalency between %HRR and %VO_2_R [3,7,21]. The current ACSM guidelines [3,7] define the threshold for moderate and vigorous intensity aerobic exercise to improve CRF as 40–59% and 60–89% of HRR or VO_2_R, respectively.

The HR–VO_2_ relationship can be influenced by the mode of exercise. Due to differences in the recruitment of muscle mass and movement patterns during exercise, one could reasonably expect to observe differences in the cardiovascular and metabolic responses to exercise between different modes of exercise, especially between weight-bearing (e.g., running) and non-weight-bearing (e.g., cycling) exercise. Findings from previous research suggest a form of exercise effect on the HRR and VO_2_R relationship [11,18,22,23]. Previous studies have evaluated the HR–VO_2_ relationship during cycle ergometry [11,16] and treadmill running [9,15,18,22,24] in only males [9,16,22], only females [24] and both males and females [11,15,18,25].

The HR–VO_2_ relationship may also be different between males and females. Although previous studies evaluating the relationship between HRR and VO_2_R included nearly equal numbers of male and female participants, the effects of sex on the HRR–VO_2_R relationship have either not been reported [18] or not presented in detail [11,15]. An earlier study [25] reported sex differences in the HR–VO_2_ relationship expressed in absolute terms (HR bpm; VO_2_ L/min).

To determine one’s HRR and VO_2_R, resting HR and VO_2_ must also be measured. Cunha et al. [26,27] described the influence of the exercise testing protocol and the manner in which resting HR and VO_2_ are measured on the HRR–VO_2_R relationship. Previous studies have measured resting values in the seated, standing or supine positions for durations ranging between 5 and 30 min [9,11,15,16,18,22,26,27]. Another study assumed a resting VO_2_ of 3.5 mL/kg/min [19]. VO_2_R is closer to HRR when a standardized protocol is used to measure resting energy expenditure [27].

Since there is a wide subject-to-subject variability in HR responses to incremental aerobic exercise [28,29], the HR–VO_2_ relationship should be individually determined when prescribing intensity of aerobic exercise. Although running and cycling are popular modes of training and are the basis of some endurance events, to the best of our knowledge no recent studies have compared the HR and VO_2_ relationship during running and cycling in the same male and female subjects when also using a standardized protocol to measure resting HR and VO_2_. Therefore, the purpose of this study was to evaluate the HR–VO_2_ relationship in males and females during treadmill running and cycling when also measuring resting HR and VO_2_ using standardized protocols.

## 2. Materials and Methods

Participants completed a resting energy expenditure study to determine resting HR and VO_2_ and two maximal graded exercise tests during two visits to the laboratory separated by 48 to 72 h. During the first visit, the resting energy expenditure study was conducted prior to performing a maximal graded exercise test on either a treadmill or an electronically braked cycle ergometer. On the second visit, the participant completed a maximal graded exercise test on the opposite mode of exercise testing that was performed during the first visit.

### 2.1. Participants

This study was reviewed and approved by the Institutional Review Board prior to the collection of any data. Forty-one (21 males; 20 females) apparently healthy adults, 18–39 years of age participated in this study. A pre-participation questionnaire was used to screen participants for known existing conditions or health issues that would limit exercise tolerance or increase the risk of untoward events during exercise. All participants provided informed written consent.

All participants met minimal physical activity recommendations for adults [1,3,4], namely, 150 to 300 min of moderate intensity physical activity per week, 75 to 150 min of vigorous aerobic exercise per week, or an equivalent combination of moderate and vigorous aerobic exercise. All participants included both running and cycling (either outdoor running and cycling or indoor running and cycling on a treadmill and a stationary bike or a combination thereof) as part of their exercise program. None of the participants were training for a specific competitive endurance event in either mode of exercise. Participants were instructed to: (a) drink plenty of fluids over the 24 h period prior to testing, (b) fast (except water) for the 8 h prior to testing, (c) refrain from the use of tobacco, alcohol, and caffeine for 4 h prior to testing, and (d) refrain from purposeful exercise during the day prior to testing.

### 2.2. Measurement of Resting and Exercise HR and VO_2_

The measurement of resting HR and VO_2_ and performance of the exercise tests occurred in the morning. Upon arrival to the lab, participants confirmed that they complied with the pre-test instructions. During the measurement of resting VO_2_ and the two graded maximal exercise tests, ventilation, expired gases, and VO_2_ were measured using a TrueOne 2400 metabolic measurement system (Parvo Medics, Sandy, UT, USA). Prior to each test, the oxygen and carbon dioxide analyzers were calibrated using medical grade gases of known concentrations and the flow meter of the metabolic measurement system was calibrated using a 3.0 L syringe at appropriate flow rates.

After measuring the height and body mass of the participant, he/she lied in the supine position on a padded table for 15 min, after which HR and VO_2_ was measured for an additional 30 min. The measurement of resting HR and VO_2_ occurred in a quiet, dim-lit room with a light blanket placed over the participant for comfort. A clear plastic dome-shaped “hood” was placed over the participant’s head. The hood was sealed by tucking its’ plastic sheet under the body or sealing the edges with towels. The hood allows for the exchange and measurement of oxygen and carbon dioxide during normal breathing without the use of a mouthpiece and nose clip. The metabolic measurement system allowed the adjustment of airflow through the system based on the carbon dioxide concentration in the hood. The metabolic measurement system was configured to calculate and display average values every minute. Resting HR was continually monitored using a radiotelemetry heart rate monitor (Polar Electro Kempele, Kempele, Finland). Resting HR and VO_2_ were calculated as the average steady-state value during the test after discarding the first 5 min of data.

After completing the resting measurements, the participant completed a maximal graded exercise test to volitional fatigue on either a treadmill (TrackmasterTMX425, Full Vision, Inc., Newton, KS, USA) or an electronically braked cycle ergometer (Lode Medical Products, Groningen, The Netherlands). Participants completed the two exercise tests in a randomized order, so half of the participants performed the running test first and half of the participants performed the cycling test first. To facilitate the measurement of oxygen consumption during exercise, participants were fitted with a one-way breathing valve, mouthpiece, and nose clip. The one-way breathing valve was held in place by a head harness. During the exercise tests, the metabolic measurement system was configured to display and print 15 s’ average breath-by-breath data. Rating of perceived exertion (RPE) was self-reported using the Borg 15-point scale [30].

In order to capture data across a full range of the HR and VO_2_ values, we began each exercise test at a relatively low workload and advanced the intensity of exercise in small increments of workload. Each stage of the exercise test was 3 min in duration. During the treadmill exercise test, the first three stages progressed from walking at a self-selected normal walking pace, a brisk walking pace, and jogging at a self-selected jogging speed on a level grade. This was followed by jogging at the same speed at a 1.5% grade and subsequently increasing the grade by 1.5% every stage until volitional fatigue occurred, at which time the exercise test was terminated. During the cycling exercise test, participants began by pedaling at a self-selected comfortable cadence between 70–90 rpm at a workload of 50 W (males and females). Each subsequent stage included increments of 20 W (females) or 25 W (males) until volitional fatigue occurred, and the subject was unable to maintain a pedal cadence of at least 50 rpm. During both exercise tests, the administrator coached the participant in order to achieve a valid maximal effort during the test. Upon reaching the point of volitional fatigue the participant grasped the hand railings on the treadmill or simply stopped pedaling during the cycling test. If performing a running test, the treadmill speed and grade were decreased to a walking speed on level grade to allow a cool down for any desired amount of time. If performing a bike test, the workload was reduced to 25–50 W and the participant pedaled at their own cadence for any desired amount of time. In both exercise tests, participant effort was considered maximal if physical signs suggestive of exhaustion were apparent and at least two of the following three criteria [7,31,32,33,34] were met: (a) maximal respiratory exchange ratio (RER) ≥ 1.10, (b) a HRmax no more than 15 bpm below age predicted maximal HR, and (c) leveling off of VO_2_ despite an increase in workload. VO_2_max during the treadmill test and VO_2_peak during the cycle test was defined as the highest average VO_2_ value recorded over any 30-s time period near the end of the test, provided RER was greater than or equal to 1.10. Maximum HR was defined as the highest HR value recorded during the test.

### 2.3. Statistical Analysis

We analyzed all data using a Bayesian approach. The initial analysis of the data included a comparison of demographic data (age, height, body mass, BMI), resting HR and VO_2_ data, maximal exercise HR (HRmax), RER, and VO_2_max or VO_2_peak data, and HRR and VO_2_R data. In some cases, we only considered sex differences (i.e., age, height, body mass, BMI, resting HR and VO_2_) and in other cases (HRmax, maximal RER, VO_2_max or VO_2_peak, HRR, and VO_2_R) we examined differences between males and females during treadmill exercise and during cycling exercise as well as differences between treadmill exercise and cycling exercise in males and in females. In all cases, mean parameters were given the same prior, and variances were given the same prior. We allowed the variance in each setting to be different (i.e., there was no constraint to impose equal variances in any of the cases). Priors for the means and variances were as follows:

μ_case_ ~ Normal (50, 10,000)

σ^2^ case ~ Gamma (1.1, 0.01)

The primary analysis of data in this study was a comparison of the intercepts and slopes of the HR–VO_2_ relationships during exercise. For each subject all 15-s interval HR and VO_2_ data were used in two models: (1) when HR and VO_2_ data were expressed as a percentage of reserve values, and (2) when HR data was expressed as a percentage of reserve values and VO_2_ data was expressed as a percentage of maximal values. Each of these two models were evaluated in the responses of men and women during treadmill running and cycle ergometry. Thus, there were eight analyses performed (2 models, 2 genders, 2 modes of exercise). Each of these analyses were accomplished using a Bayesian hierarchal model to appropriately account for multiple measurements for each individual. In our framework, each subject’s linear response is considered to be a deviation from an overall population linear response. Although we actually estimate the intercept and slope parameters for each individual, it is these population parameters for intercept and slope that are of primary interest. We used fairly noninformative priors for the population parameters. All posterior inference was performed on the posterior distributions of the population parameters. For each of the eight relationships we described above, we had the following set up relative to the HR measurement. (In the text that follows, the normal distribution is noted as N (mean, variance), and the gamma distribution is noted as Gamma (shape, rate; i.e., the mean of the gamma is shape/rate.)

HR_i ~_ Normal (μ_i_, σ^2^)

μ_i_ = b0_id_ + b1_id_ * VO_2i_

The intercepts (b0_id_) and slopes (b1_id_) are estimated for each individual (as identified by the id). The prior distributions for the parameters in each case for each individual were as follows:

b0_id ~_ Normal (μ_b0_, σ^2^_intercepts_)

b1_id ~_ Normal (μ_b1_, σ^2^_slopes_)

The prior distributions for the population parameters were fairly noninformative and were:

μ_b0 ~_ Normal (0, 1000)

μ_b1 ~_ Normal (1, 10)

The prior distributions for the variance components were as follows:

σ^2 ~^ Gamma (1.1, 0.01)

σ^2^_intercepts ~_ Gamma (1.1, 0.01)

σ^2^_slopes ~_ Gamma (1.1, 1)

Thus, we appropriately accounted for all sources of variation, including random error, and error due to the individual intercepts and slopes.

Code was run in R [35] using JAGS (Just Another Gibbs Sampler) [36]. Since complicated Bayesian models rely on Markov chain Monte Carlo (MCMC) methods it is important to consider convergence diagnostics of the MCMC samples. Each chain of the models was run using 1000 burn in iterations and 11,000 total iterations. We used 20 chains and thinned our output by 20 for each run, yielding a total of 10,000 saved iterations for each estimated parameter. The Rhat statistic was computed for each posterior chain [37] and in each case was less than 1.002. The Raftery-Lewis statistic [38] was computed for each chain and never exceeded 1.05. Effective sample sizes all exceeded 8600. All posterior convergence diagnostics were computed using the R package, coda [39]. All inferences were made using posterior distributions of the population parameters.

In the analysis of age, height, weight, BMI, resting HR and VO_2_, maximal HR, RER and VO_2_, and HRR and VO_2_R, differences between males and females or differences between treadmill exercise and cycling exercise were considered significant if zero (0) fell outside of the 95% credible interval (CI) and the posterior probability of the difference being greater than zero was greater than or equal to 95%.

In the analysis of HR and VO_2_ relationship, VO_2_ was considered the independent variable and HR was considered the dependent variable. We were interested in determining if the intercept and slope of the line of best fit through the data of each of the HR–VO_2_ relationships were different from zero (0) and one (1), respectively. Means, standard deviations, and posterior probability intervals, or CI, were generated for the slope and intercept of each of the eight models. The intercept was considered significantly different from zero (0) if zero was outside the 95% CI. The slope was considered significantly different from one (1) if one was outside the 95% CI.

We also compared the intercepts and slopes of each HR–VO_2_ relationship in males and females between the two modes of exercise, as well as within each mode of exercise. The intercept and slope of each HR–VO_2_ relationship in females was considered significantly different than that of males if the posterior probability exceeded 95%.

We estimated the mode and 95% credible intervals for %HRR values at 45%, 55%, 65%, 75% and 85% of VO_2_max and VO_2_R values. We considered the %HRR point estimate to be significantly different from the same %VO_2_max or %VO_2_R value if the estimated %VO_2_max or %VO_2_R value fell outside of the CI of the %HRR.

## 3. Results

Table 1 includes some of the personal characteristics of the participants in the study. Table 2 includes results from the REE and maximal exercise test results. Resting HR was significantly higher in females. Resting VO_2_ was significantly higher in males. Males achieved a significantly higher maximal RER than females during treadmill running but not cycling. There were no sex differences in maximal HR or HRR during treadmill running or cycling. Males achieved significantly higher VO_2_max (or VO_2_peak) and VO_2_R values than females during both treadmill running and cycling. There were no significant differences in maximal HR, VO_2_max (or VO_2_peak), HRR, and VO_2_R between treadmill running and cycling in either males or females.

Table 3 includes the intercepts and slopes for the regression lines for the %HRR–%VO_2_max and %HRR–%VO_2_R relationships for males and females during treadmill running and cycling. The intercepts and slopes of the %HRR–%VO_2_max and %HRR–%VO_2_R relationships during running and cycling in both males and females were all significantly greater than an intercept of zero (zero lies outside the CI) and less than a slope of 1 (1 lies outside the CI), respectively.

There were no significant differences in the intercepts of the %HRR–%VO_2_max and %HRR–%VO_2_R relationships between males and females (probability of a sex difference < 95%) during running and cycling, or between running and cycling in either males or females (probability of a mode difference < 95%). During running, the slopes of the %HRR–%VO_2_max and %HRR–%VO_2_R relationships were significantly greater in females than in males (probability of a sex difference > 95%).

During cycling, there were no significant differences in the slopes of the %HRR–%VO_2_max and %HRR–%VO_2_R relationships between males and females (probability of a sex difference < 95%). There were no significant differences in the slopes of the %HRR–%VO_2_max and %HRR–%VO_2_R relationships between treadmill running and cycling in either males or females (probability of a mode difference < 95%).

Table 4 includes the modal %HRR value and credible interval at a range of moderate- to vigorous-intensity %VO_2_max and %VO_2_R values (45%, 55%, 65%, 75%, 85%) recommended by the ACSM [3,7] to improve CRF. The %HRR significantly overestimated %VO_2_R at all intensities less than 85% of VO_2_R during treadmill running and cycling for both males and females. The %HRR overestimated %VO_2_R more so at lower intensities of exercise than at higher intensities of exercise. The %HRR significantly overestimated %VO_2_max only at intensities of exercise representing 45% and 55% of VO_2_max during treadmill running and cycling for males. In females, %HRR significantly overestimated %VO_2_max only at intensities of exercise representing 45% and 55% of VO_2_max during treadmill running and 45%, 55% and 65% of VO_2_max during cycling.

## 4. Discussion

This study is unique in that we used Bayesian statistics to evaluate the HR–VO_2_ relationship in a group of males and females during two modes of exercise, treadmill running and stationary bike cycling.

### 4.1. HR–VO_2_ Relationship

A key finding in this study was that neither the %HRR–%VO_2_max or %HRR–%VO_2_R relationships coincided with the line of identity (intercept = 0; slope = 1) in either males or females during cycling or treadmill exercise (Table 3). Although neither relationship coincided with the line of identity, the %HRR–%VO_2_max relationship was closer to the line of identity than the %HRR–%VO_2_R relationship (Table 4). The results of this study concur with three previous studies that have reported similar results during treadmill [18] and cycling [20,40] exercise. On the contrary, other studies reported that the %HRR–%VO_2_R relationship was not distinguishable from the line of identity, but the %HRR–%VO_2_max relationship did not coincide from the line of identity during treadmill [15] and cycling [11,16] exercise. Swain et al. suggested that the differences in the HR–VO_2_ relationship they reported during cycling [11] and treadmill [18] exercise in the same lab could be attributed to the mode of exercise. Nevertheless, the subjects in the two studies [11,18] were not the same. Previous studies have evaluated the HR–VO_2_ relationship during treadmill [15,18,24] or cycling [11,16,20,40] exercise. To the best of our knowledge, this may be the first study that used the same group of subjects to evaluate the HR–VO_2_ relationship during both treadmill and cycling exercise. Our data, and that of three previous studies [18,20,40] that report an inequality in the %HRR–%VO_2_max and %HRR–%VO_2_R relationships add support to the need to rethink the HR–VO_2_ relationship and its implications for exercise prescription.

Several factors may contribute to the disagreements in the data reported between this and previous studies. Differences in how resting HR and VO_2_ are measured may contribute to differences in the calculation of HRR and VO_2_R and therefore the %HRR–VO_2_R or %HRR–%VO_2_max relationship. In this study, we followed the recommendations for measuring resting energy expenditure, namely ensuring a minimum of 10–20 min rest before the assessment, followed by a 25–30 min measurement duration, discarding the first 5 min [26]. Resting HR and VO_2_ have been measured during 5 min of seated rest [11], during the last 2 min of a 5 min seated rest period [18], and during the last 5 min of a 20 min seated rest period [16]. Following a 10 min rest period, resting values have been measured for 30 min in the supine position, discarding the first 20 min [15].

The measurement of HR and VO_2_ during exercise may also affect the HR–VO_2_ relationship. In this study, we programmed our metabolic measurement system to average data every 15 s. The number of data points for each subject varied with the duration of the exercise test. For the 41 subjects, we had 4184 HR–VO_2_ data points (an average of approximately 51 data points for each exercise test for each subject). Other studies averaged HR and VO_2_ data during the last 30 s [18] or during the last minute of each stage [11,15,16].

One cannot assume an equivalency of the HR–VO_2_ relationship between individuals. Training status or cardiovascular fitness level may also affect the HR–VO_2_ relationship during exercise. For example, Swain et al. [11,18] reported a fitness effect in the %HRR–%VO_2_max relationship in that the individual regression lines for less-fit participants had a greater intercept than for the high-fit participants. The implications of this is that an exercise prescription based on the assumed equivalency of the %HRR–%VO_2_max relationship would produce greater errors for low-fitness individuals. Lounana et al. [16] reported that the regression for the %HRR–%VO_2_max relationship did not coincide with the line of identity, but the regression for the %HRR—%VO_2_R was indistinguishable from the line of identity in elite male (average VO_2_max = 70.9 ∀ 1.2 mL/kg/min) road cyclists.

### 4.2. Sex Differences in the HR-VO_2_ Relationship

Despite sex differences in resting HR and VO_2_, VO_2_max, and VO_2_R (Table 2), (a) there were no sex differences in the intercepts of the %HRR–VO_2_R or %HRR–%VO_2_max relationship during treadmill running or during cycling (Table 3); (b) there were no differences in the intercepts between running and cycling in males or in females (Table 3); (c) there were no sex differences in the slopes during cycling; and (d) the slopes were significantly greater in females than in males during treadmill running. Our data is in agreement with that of previous studies that reported no differences in the intercepts or slopes of the regression lines between males and females during cycling [11] and during treadmill [15] exercise. Another study did not report an analysis of the data by sex during treadmill running [18]. In addition to the other factors that affect the HR–VO_2_ relationship, one cannot presume that the relationship is equivalent in males and females. Future research can include both male and female participants, analyze data for sex differences, and report differences in the HR-VO_2_ relationship between males and females.

### 4.3. Implications for Exercise Prescription

The ACSM currently suggests prescribing the intensity of aerobic exercise based on the %HRR–%VO_2_R relationship [3,7,21]. This is supported by previous studies that have reported equality in the %HRR–%VO_2_R [11,15,16] relationship. Assuming the regression line representing the %HRR–%VO_2_R relationship coincides with the line of identity (intercept = 0, slope = 1), a given %HRR would approximate the same %VO_2_R. Thus, using a %HRR value to prescribe the intensity of aerobic exercise would provide an accurate estimate of the metabolic cost of the exercise. Basing an exercise prescription on %VO_2_R (rather than %VO_2_max) more accurately reflects the net energy expenditure of the exercise, which is more useful in exercise programs that include a weight loss intention [18].

We report an inequality between the line of identity and the %HRR–%VO_2_R and the %HRR–%VO_2_max relationships. Our data is in agreement with data previously reported during cycling [20,40] and treadmill exercise [18]. Our data supports the need to rethink the HR–VO_2_ relationship and its accepted use for prescribing intensity of exercise–especially for an individual. The HR–VO_2_ relationship is influenced by ambient and body temperature, hydration, training status, emotional state and the day-to-day variations in resting HR and HR responses to exercise [40]. The wide CI reported in this study for the slopes and intercepts (Table 4) reflects the interindividual variability in the HR and VO_2_ responses during exercise. Understanding the potential variability in the intercepts and slopes of the HR–VO_2_ relationship, but assuming an equivalency between %HHR and %VO_2_max or %VO_2_R, can introduce error when prescribing intensity of exercise for an individual. The efficacy of prescribing intensity of steady-state, constant-load aerobic exercise, using the HR–VO_2_ relationship established during incremental intensities of exercise, is also controversial [40].

The ACSM [3,7] suggests a threshold for moderate intensity aerobic exercise (40–59% of HRR or VO_2_R) and vigorous intensity aerobic exercise (60–89% of HRR or VO_2_R) to improve CRF. A review of previous studies supports the use of 45% of VO_2_R as a minimal effective training intensity to improve CRF [10]. In this study, 45% and 55% HRR did not accurately estimate the corresponding %VO_2_R or %VO_2_max during treadmill running or cycling (Table 4). At higher intensities, 65%, 75% and 85% of HRR accurately estimated the corresponding %VO_2_max (but not VO_2_R) during treadmill running and cycling in men and treadmill running in women. Thus, it appears that the %HRR–%VO_2_max relationship approaches equality as intensity of exercise increases. This may be due to a greater variability in the HR responses to exercise at lower intensities. Based on the data reported in this study (Table 4), %HRR does not accurately estimate %VO_2_R at intensities of exercise below 85% of VO_2_R in both males and females during treadmill running or cycling.

### 4.4. Study Limitations

This study has several limitations. The participants in this study included college coeds ranging in age between 18 and 39 years of age. All participants included running and cycling as part of their exercise program. The time spent in each mode of training was not likely equal for each participant and quite variable between participants. The results of this study may not apply to other age groups or to individuals with more experience in either running or cycling. The results of a recent study [41] indicated that endurance-trained trail runners achieved similar VO_2_max values but lower maximal HR values during a treadmill exercise test that progressed in increments of incline, compared with one that progressed in increments of speed. The reported results [41] may suggest that the responses to a treadmill test that increases in speed or incline may reflect the type of training typically performed by the participant. The HR–VO_2_ relationships observed in this study may be influenced by the nature of the exercise test performed.

## 5. Conclusions

The %HRR–%VO_2_R and the %HRR–%VO_2_max relationships during treadmill running and cycling in men and women are significantly different from the line of identity, and %HRR is more closely associated with %VO_2_max than to %VO_2_R. The %HRR accurately estimates %VO_2_max at intensities above 55% of VO_2_max. The variability in the intercepts and slopes of the HR–VO_2_ relationships between individuals can lead to error in prescribing intensity of exercise for an individual. The inequality between %HRR and either %VO_2_max or %VO_2_R during treadmill running and cycling in males and females adds to the debate on the use of these relationships to prescribe intensity of aerobic exercise.

## Figures and Tables

**Table 1:** Participant characteristics.

	Male (*n* = 21)	Female (*n* = 20)
Age	25.2 ± 5.4	22.0 ± 5.2
Body Mass (kg) ^a^	72.9 ± 10.8	58.8 ± 6.8
Height (cm) ^a^	180.6 ± 9.5	163.8 ± 5.7
BMI (kg/m^2^)	22.3 ± 2.4	21.9 ± 2.3

^a^ Significant difference between males and females (posterior probability > 95%).

**Table 2:** Resting and maximal exercise test results.

	Male (*n* = 21)	Female (*n* = 20)
Resting		
Resting HR (bpm) ^a^	53.9 ± 6.3	59.1 ± 7.7
Resting VO_2_ (mL/kg/min) ^a^	3.7 ± 0.35	3.4 ± 0.37
Treadmill maximal exercise test		
Maximal RER ^a^	1.20 ± 0.09	1.14 ± 0.08
Maximal HR (bpm)	183.0 ± 11.9	188.6 ± 12.4
Maximal VO_2_ (mL/kg/min) ^a^	57.3 ± 7.1	45.7 ± 3.3
Heart rate reserve (bpm)	129.1 ± 13.0	129.5 ± 13.9
VO_2_ reserve (mL/kg/min) ^a^	53.6 ± 7.0	42.3 ± 3.0
Cycling maximal exercise test		
Maximal RER	1.20 ± 0.06	1.18 ± 0.05
Maximal HR (bpm)	180.0 ± 11.5	182.7 ± 12.1
Peak VO_2_ (mL/kg/min) ^a^	54.5 ± 7.1	43.7 ± 5.8
Heart rate reserve (bpm)	126.1 ± 12.6	123.6 ± 13.5
VO_2_ reserve (mL/kg/min) ^a^	50.7 ± 7.0	40.3 ± 5.6

^a^ Significant difference between males and females (posterior probability > 95%).

**Table 3:** Bayesian analysis of the HR–VO_2_ relationship.

	Treadmill Running		Cycling	
	Males	Females	Males	Females
%HRR vs. %VO_2_max				
Intercept	12.42 ± 2.24	8.44 ± 2.27	9.99 ± 1.87	10.78 ± 2.02
Credible interval	(8.00, 16.88)	(3.98, 12.96)	(6.29, 13.64)	(6.78, 14.80)
Slope	0.876 ± 0.023 *	0.943 ± 0.024 *	0.912 ± 0.018	0.931 ± 0.021
Credible interval	(0.831, 0.921)	(0.895, 0.990)	(0.876, 0.947)	(0.890, 0.973)
%HRR vs. %VO_2_Res				
Intercept	18.26 ± 2.09	15.38 ± 2.11	16.38 ± 1.83	18.06 ± 1.9
Credible interval	(14.02, 22.4)	(11.2, 19.54)	(12.78, 20.0)	(14.19, 21.92)
Slope	0.818 ± 0.021 *	0.873 ± 0.023 *	0.849 ± 0.017	0.858 ± 0.019
Credible interval	(0.777, 0.858)	(0.828, 0.918)	(0.815, 0.884)	(0.821, 0.896)

Values are intercepts and slopes ± SD (Credible interval). * = Significant sex difference in slopes within the mode of exercise (probability that the slope for females is greater than the slope for males is >95%).

**Table 4:** %HRR at designated values of %VO_2_max or %VO_2_R values.

	Percent VO_2_max or VO_2_R				
	45%	55%	65%	75%	85%
%HRR vs. %VO_2_max—MEN					
Treadmill running	52% *	61% *	69%	78%	87%
	(47.1, 56.7)	(55.7, 65.6)	(64.3, 74.6)	(72.8, 83.6)	(81.3, 92.7)
Cycling	51% *	60% *	69%	78%	87%
	(47.1, 55.0)	(56.1, 64.3)	(65.0, 73.6)	(73.9, 82.8)	(82.8, 92.2)
%HRR vs. %VO_2_max—WOMEN					
Treadmill running	51% *	60% *	70%	79%	89%
	(46.0, 55.7)	(55.2, 65.3)	(64.4, 74.9)	(73.6, 84.6)	(82.7, 94.3)
Cycling	53% *	62% *	71% *	81% *	90%
	(48.4, 57.0)	(57.5, 66.4)	(66.6, 75.9)	(75.7, 85.5)	(84.8, 95.1)
%HRR vs. %VO_2_R—MEN					
Treadmill running	55% *	63% *	71% *	80% *	88%
	(50.6, 59.5)	(58.6, 67.8)	(66.5, 76.2)	(74.6, 84.6)	(82.5, 93.1)
Cycling	55% *	63% *	71% *	80% *	88%
	(50.7, 58.4)	(59.1, 67.0)	(67.5, 75.7)	(75.7, 84.4)	(84.1, 93.1)
%HRR vs. %VO_2_R—WOMEN					
Treadmill running	55% *	63% *	72% *	81% *	90%
	(50.2, 59.2)	(58.8, 68.1)	(67.3, 77.1)	(75.7, 86.1)	(84.2, 95.1)
Cycling	57% *	65% *	74% *	82% *	91% *
	(52.7, 60.8)	(61.1, 69.5)	(69.5, 78.3)	(77.9, 87.1)	(86.2, 95.9)

* Point estimates of %HRR are significantly different from the corresponding %VO_2_max or %VO_2_R.

## Data Availability

The data presented in this study and the R and JAGS code are available upon request from the corresponding author. The data are not publicly available.
